# Functional constipation in children: does maternal personality matter?

**DOI:** 10.1186/1824-7288-35-25

**Published:** 2009-08-12

**Authors:** Alireza Farnam, Mandana Rafeey, Sara Farhang, Saeedeh Khodjastejafari

**Affiliations:** 1 Research team for psychiatry and behavioral sciences, Liver and gastrointestinal diseases research center, Tabriz University of medical sciences, East Azerbaijan, Tabriz, Iran

## Abstract

**Aim:**

To identify personality dimensions of mothers with a constipated child and compare it with those mothers of children without defecation problems.

**Methods:**

We compared mothers of 150 children with functional constipation to mothers of 150 children with no such a problem attending to pediatric hospital of Tabriz University of medical sciences. Personality dimensions were evaluated by NEO five factor inventory after excluding any psychiatric disorders by an interview.

**Results:**

Mean age (SD) was 28.8(18.6) months in constipated children and 20.0(19.3) months in controls, 54.6% of constipated children and 56.7% of controls were male. Mean age (SD) was 30.9(7.1) years in mothers of children with functional constipation and 30.1(7.6) years in controls.

Mothers of children with functional constipation scored lower in neuroticism and scored higher in extraversion, conscientiousness and agreeableness. Conscientiousness was the dominant dimension of personality in both groups.

**Conclusion:**

Our results suggest the maternal personality as a factor to directly influence toileting behavior of their children resulting in functional constipation.

## Aim

The non-organic Childhood constipation is a widespread problem and a recent systematic review has estimated the prevalence to be 0.7% to 29.6% [1]. A multifactorial pathophysiology is more accepted among researchers. Low fiber intake, psychiatric factors and positive family history [2-4] as well as experiencing stressful events in family and instability in the child-parent relationships are the reported explanations [5]. The stool-withholding behavior is known to be the major cause for the development and/or persistence of constipation in childhood [6]. Defining the associated psychiatric factors will improve the challenging treatment especially in chronic and recurrent situations.

The parental behavior strongly influences the mental and physical situation of their children such as they may have an effect on the manifestations of a disorder. Functional abdominal pain in children has been described to be increased by more "attention" from their parents [7]. Again, they are the ones who decide help seeking and influence the treatment decisions. Training the parents is an important part of the standard pediatric care for children with functional constipation [8]. The mother may be the parent to play the most important role in early childhood. Studies report that secure attachment and maternal secure base support are related to higher levels of positive mood, more constructive coping and better regulation of emotion [9]. However; the mother-child relationship has not been studies in details concerning its effect on defecation behavior of the children. The parenting style of a mother is defined by her personal characteristics. We hypothesized that the control on defecation in a child is influenced by the common manners of mother; e.g. her personality.

The aim of this study is to examine the relations between the personality dimensions in mothers and existence of the functional constipation in their children.

## Methods

Study population was recruited continuously from the university pediatric clinic (Tabriz University of medical sciences, Iran) during September 2007–September 2008. All of the patients with the complaint of constipation were fully evaluated by a same pediatric gastroenterologist and children with functional constipation (based on ROME III criteria for pediatric functional constipation) were enrolled in the study. Another group including mothers of children attending to the same clinic and without functional constipation was invited to participate as the control group. Mothers then were invited for a psychiatric assessment. A history and/or current symptoms related to psychiatric disorders on Axis I (based on DSM-IV) [10] or seizure disorders in mothers led to exclusion from the study. The research procedure was compatible with Helsinki Declaration and all of the participants gave written consent.

Personality dimensions in both groups were evaluated by the NEO Five-Factor Inventory (NEO-FFI) which provides a dimensional account of the structure of normal personality traits, dividing the personality into five broad dimensions which are: extraversion, agreeableness, conscientiousness, neuroticism and openness to experience [11]. This 60-item standard questionnaire usually requires 15 minutes to complete and is rated on a five-point scale to yield scores in five major domains of personality.

Means (standard deviations) were used to describe continuous variables and proportions for categorical data. Conditions were met for using Two-tailed Student's t test and Chi-square test, which was applied when appropriate and the overall significance was set at 0.05.

## Results

One hundred and fifty children were evaluated in each group. No significant difference was noted between the two groups regarding their age (p = 0.240) and gender (p = 0.354). Mean age (SD) was 28.8(18.6) months in constipated children and 20.0(19.3) months in controls, 54.6% of constipated children and 56.7% of controls were male.

Mean age (SD) was 30.9(7.1) years in mothers of children with functional constipation and 30.1(7.6) years in controls (p = 0.348). In both groups; near to 50% had a diploma degree, about 30% were educated under diploma and about 20% were postgraduates (p = 0.777).

**Figure 1:**
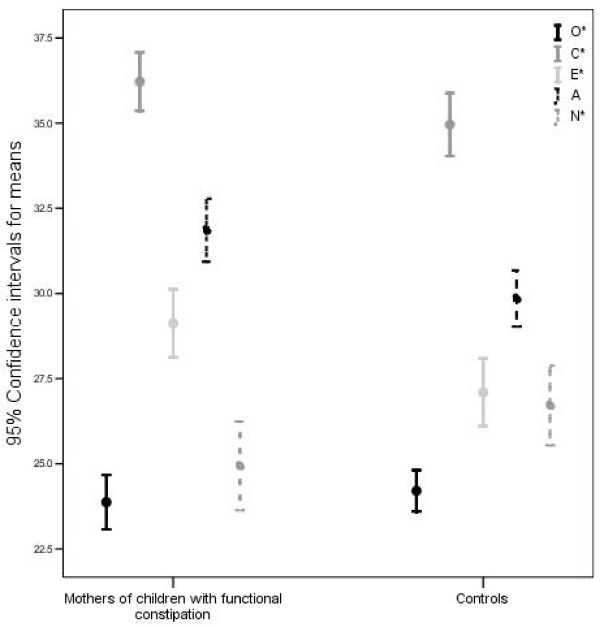
**Error bars comparing scores of five personality dimensions in mothers of children with functional constipation and controls**. Personality of mothers of children with functional constipation was assembled of significantly lower neuroticism and higher extraversion, conscientiousness and agreeableness compared to mothers of children without functional constipation.

The scores of five personality dimensions assessed by NEO inventory are described in figure 1 by error bars. These two groups had significant differences. Mothers of children with functional constipation scored lower in neuroticism [(24.9(8.1) vs. 26.7(7.2), p = 0.046] and scored higher in extraversion [29.1(6.2) vs. 27.1(6.2), p = 0.005], conscientiousness [36.2(5.3 vs. 34.9(5.7), p = 0.049] and agreeableness [31.9(5.7) vs. 29.8(5.1), p = 0.002]. No significant difference was observed in the score of openness to experiences [23.9(4.9) vs. 24.2(3.7), p = 0.512].

We also compared two groups according to their dominant personality dimension. The dominant personality dimension was not statistically different between the two groups. The most common was conscientiousness (58.5%) followed by neuroticism (18.1%).

## Discussion

Studies have documented the associations between motility disorders of the gastrointestinal tract (like chronic constipation) and psychological stresses like anxiety and depression [12,13]. We have previously reported specific overstated personality dimensions and the personality profile to be similar in constipation dominant variant of irritable bowel syndrome [14]. Likewise; the basis of pediatric functional constipation may be explained by psychoanalysis of the family events and relationships. Parents of children with idiopathic constipation may have no psychological problems [15] however cultural and social pressures are described to result in constipation in children as an "over-control problem" [16]. According to these results; we suggested and checked up the personality variations of mothers; which are not a "disorder" but may influence the child-mother relationship and child's behavior.

The frequency of "hiding to stool" and "asking for pull-ups" in constipated children indicates full bowel control and their social awareness [17] so maternal expectations may design her child's reactions. While the extreme variation of a personality dimension (personality disorder) [18] in mothers was excluded by an interview, we believe that this study could trace the differences between them (extent of each dimension) in a way which is describing their manner and relationships. This study benefited using the five-factor model of personality which is considered to be the most comprehensive experimental enquiry into personality.

Our results generally supported a personality difference between mothers with or without constipation in their children. Lower score in neuroticism, besides higher score in extraversion, conscientiousness and agreeableness is compatible with a character described by forcefulness, dutifulness, self conscientious, orderliness and discipline who is optimist, proficient, inflexible with less humiliation, anxious and restlessness. This restriction, force and orderliness may result in resistance and withholding as a response of child to the maternal behavior. This response may demonstrate a messy, careless or destructive character against the inflexible approach of the mother up to an orderly, rigid and obsessive personality.

In conclusion, a considerable personality was obvious in mothers of constipated children in the current study which is compatible with a character that may rise up the stool-withholding behavior in their children. Future researches must include personality of mothers as a noteworthy factor in evaluating the treatment options.

## Authors' contributions

AF had primary responsiblity for protocol development, patient screening, enrollment, outcome assessment. MR and SK participated in the development of the protocol and were responsible for patient screening. SF supervised the design and execution of the study, performed the data analyses and writing the manuscript. All authors have read and approved the final manuscript.
